# Polysaccharides as a Hydrophilic Building Block of Amphiphilic Block Copolymers for the Conception of Nanocarriers

**DOI:** 10.3390/pharmaceutics16040467

**Published:** 2024-03-27

**Authors:** Aijing Lu, Suming Li

**Affiliations:** 1 NMPA Key Laboratory for Quality Research and Control of Tissue Regenerative Biomaterial & NMPA Research Base of Regulatory Science for Medical Devices, Institute of Regulatory Science for Medical Devices, Sichuan University, Chengdu 610064, China; 15680815509@163.com; 2 Institut Européen des Membranes, UMR CNRS 5635, Université de Montpellier, 34095 Montpellier, France

**Keywords:** polysaccharide, block copolymer, amphiphilic, nanocarriers, drug delivery system

## Abstract

Polysaccharides are gaining increasing attention for their relevance in the production of sustainable materials. In the domain of biomaterials, polysaccharides play an important role as hydrophilic components in the design of amphiphilic block copolymers for the development of drug delivery systems, in particular nanocarriers due to their outstanding biocompatibility, biodegradability, and structural versatility. The presence of a reducing end in polysaccharide chains allows for the synthesis of polysaccharide-based block copolymers. Compared with polysaccharide-based graft copolymers, the structure of block copolymers can be more precisely controlled. In this review, the synthesis methods of polysaccharide-based amphiphilic block copolymers are discussed in detail, taking into consideration the structural characteristics of polysaccharides. Various synthetic approaches, including reductive amination, oxime ligation, and other chain-end modification reactions, are explored. This review also focuses on the advantages of polysaccharides as hydrophilic blocks in polymeric nanocarriers. The structure and unique properties of different polysaccharides such as cellulose, hyaluronic acid, chitosan, alginate, and dextran are described along with examples of their applications as hydrophilic segments in the synthesis of amphiphilic copolymers to construct nanocarriers for sustained drug delivery.

## 1. Introduction

Nanotechnology has emerged in the past decades as a ubiquitous technology with diversified applications in different fields ranging from industry to healthcare. In the latter field, nanomedicine has recently attracted growing interest for applications in medical imaging, disease diagnosis, drug delivery, etc. [1]. In particular, nano-sized drug delivery systems have been extensively investigated because of their outstanding properties such as good stability and high drug load and their feasibility of incorporating both hydrophobic and hydrophilic molecules, as well as various drug administration routes (inhalation, oral, topical, and parenteral injection) [1,2,3,4,5,6]. Moreover, nanocarriers enable to improve the drug bioavailability, to diminish the dosing frequency, to reduce the side effects, and to achieve targeted drug delivery [7,8].

The toxicity of drugs remains a major challenge for developing new and safe drugs as it may affect the biological processes involving a single target organ or multiple organs [1,9]. Among the different target organs, the liver, kidney, heart, and central nervous system are the most commonly observed ones for clinical drug development. Compared to small-molecule drugs, nanocarriers exhibit slower kinetic transport into diseased tissues due to vascular barriers. Therefore, a relatively extended circulation time of nanomaterials is essential to improve the likelihood of crossing the vascular wall. The stealth effect, which is the guarantee of long circulation, significantly enhances the pharmacokinetics, including blood circulation, biodistribution, and tissue targeting, thereby allowing for drug delivery applications of nanomaterials [9]. Various nanocarriers have been developed to encapsulate drugs, including nanoparticles [10,11], nanogels [12], liposomes [13], and micelles [14] prepared from lipids, polymers, and inorganic materials. In this way, drug delivery systems could reduce the side effects of drugs and improve treatment efficiency. Hybrid nanocarriers have also been designed to take advantage of both polymers and inorganic materials [15]. Similarly, inorganic nanoparticles have been prepared to deliver drugs to the tumor site to improve drug effectiveness [5]. Nevertheless, polymeric drug delivery systems have attracted more attention because their sizes, geometries, surface properties, and structures can be tailored by varying the chemical composition and topology of the polymers which compose the systems [16]. Several types of polymers have demonstrated great potential for clinical applications, including natural polymers and synthetic polymers [17]. PEG is the most widely used synthetic polymer in clinical applications, and the marketed products include Adagen^®^, Oncospar^®^, PegIntron^®^, Neulasta^®^, and Somavert^®^. Several other types of polymers are also used in clinics, such as poly(hydroxypropyl methacrylamide) (ProLindac^TM^) and cyclodextrin-based polymers (CALAA-01^®^, IT-101^®^) [17].

Aliphatic polyesters have attracted much attention in recent decades as drug delivery systems due to their outstanding biodegradability, biocompatibility, and versatility regarding their physical, chemical, and biological properties [18]. The in vivo degradation of the polyester backbone and bioresorption of degraded products prevent accumulation of the drug carrier in the body, thus reducing the risk of long-term toxicity. Common polyesters used in drug delivery applications include polylactide (PLA) [19], poly(ε-caprolactones) (PCL) [20], polyglycolide (PGA) [21], polydioxanone (PDO) [22], and poly(lactide-co-glycolide) (PLGA) [23]. A number of products are currently used in clinics in the form of microspheres (Lupron Depot^®^, Eligard, Risperdal Consta, Trelstar LA, and Sandostatin LAR), implants (Durin^®^), and hydrogels (Oncogel^®^). Nevertheless, synthetic polymers generally present poor biological activities [18].

Natural polymers or biopolymers are macromolecules that are obtained from plants, animals, and microorganisms. Polysaccharides, including cellulose, chitin or chitosan, hyaluronate, and alginate, are the most abundant biopolymers composed of a large number of monosaccharides linked together by O-glycosidic linkages [24]. These biopolymers present intrinsically beneficial properties such as antioxidant, antiviral, anticoagulant, anticancer, and immune-modulating activities [25], and have been largely used in the preparation of drug delivery systems such as hydrogels, nanogels, nanoparticles, micelles, and drug conjugates due to their outstanding biocompatibility, biodegradability, and inherent biological properties [24,26]. Nevertheless, the utilization of native polysaccharides as drug carriers is limited due to their poor solubility and processability. In contrast, their derivatives of relatively low molecular weights have been attracting much interest as a hydrophilic building block of amphiphilic block copolymers which can be used for the development of various nanocarriers. In this review, we will present the state of the art of polysaccharide-based nanocarriers for uses in drug release, including the synthesis methods of amphiphilic block copolymers involving a hydrophilic polysaccharide block and different polysaccharides used in the formulation of nanocarriers. 

## 2. Synthesis Methods to Prepare Polysaccharide-Based Block Copolymers 

**Figure 1:**
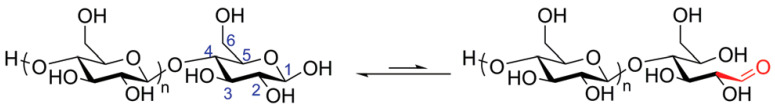
Illustration of ring-closed and ring-opened forms of a cellulose reducing end.

A polysaccharide is a long polymeric chain containing a large number of functional groups, including secondary or primary hydroxyl, amine, and carboxyl groups with different reactivities. Various amphiphilic copolymers have been designed via grafting hydrophobic polymers onto the functional groups of polysaccharide backbone or via polymerization initiated by the functional groups [27]. However, such reactions are poorly controllable and may lead to complex structures. In contrast, the presence of a reducing end on polysaccharide chains makes it possible to synthesize block polymers. As shown in Figure 1, there exists an equilibrium between ring-closed and ring-opened forms in one of the two polysaccharide chain ends, also called the reducing end. The opened form has an aldehyde group which is available for further reactions with other functional groups to synthesize block copolymers, such as chain extension, coupling, and other types of reactions.

### 2.1. Reductive Amination

Reductive amination is a widely employed technique in organic synthesis due to its selectivity, generally rapid reaction kinetics, and straightforward execution. This two-step process entails the condensation of a primary amine with an aldehyde, resulting in the formation of an imine intermediate. Typically, a reducing agent like sodium borohydride is incorporated, which promptly and selectively reduces the imine as it forms, yielding a secondary amine. Maintaining control over the pH level is crucial in this procedure. A pH range of 6 to 8 is considered optimal for most reductive amination reactions [28,29].

**Figure 2:**
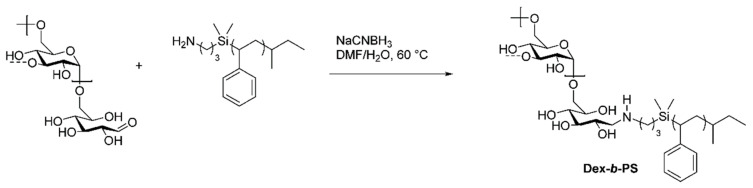
Synthesis of dextran-*b*-PS diblock copolymer via reductive amination [32].

Reductive amination has been extensively employed for the functionalization of polysaccharides because the reducing end of polysaccharides is available for this reaction [30]. The first applications of this reaction in polysaccharide chemistry consist in coupling small molecules and proteins to alginate, cellulose, and dextran to yield solid matrices for use in affinity chromatography [31]. In 2002, this approach was adopted by Bosker et al. to prepare block copolymers of dextran (Dex) and polystyrene (PS) with a molecular weight of 12 kg/mol [32]. These block copolymers were prepared via reductive amination between the terminal primary amines present in PS chains and the C1 aldehyde groups located at the reducing end of dextran, as depicted in Figure 2.

**Figure 3:**
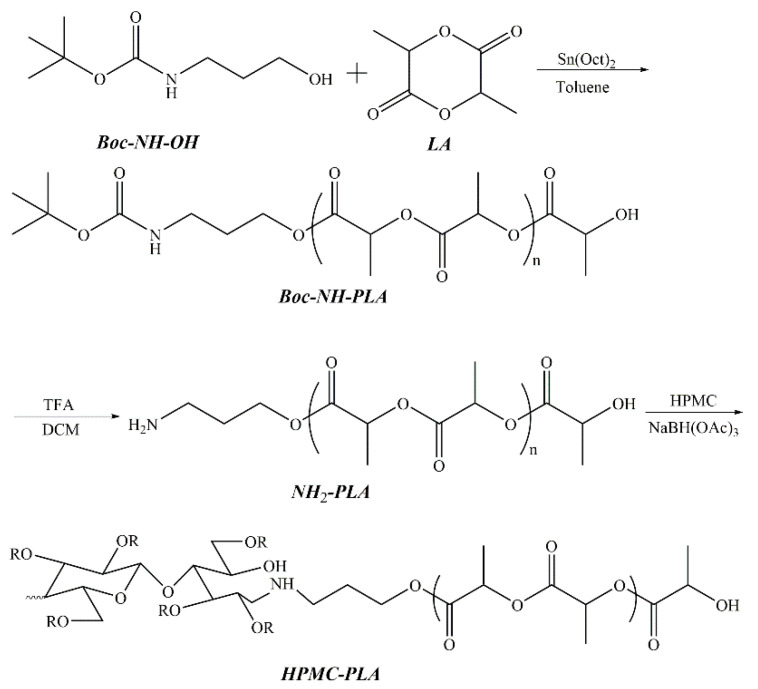
Synthesis route of HPMC-PLA diblock copolymers by reductive amination [33].

Lu et al. reported the synthesis of a series of amphiphilic block copolymers from hydroxypropyl methyl cellulose (HPMC) and amino-terminated polymers, including HPMC-PLA [33], HPMC-Jeffamine [34], and HPMC-PCL [35]. Figure 3 shows the synthesis route of HPMC-PLA diblock copolymer using a three-step procedure. First, chain-end-protected PLA (Boc-NH-PLA) was synthesized by the ring-opening polymerization (ROP) of lactide initiated by tert-butyl-N-(3-hydroxypropyl) carbamate. The N-tert-butoxycarbonyl (Boc) group was then removed from Boc-NH-PLA using trifluoroacetic acid (TFA). Finally, HPMC-PLA copolymers with different PLA block lengths were synthesized via reductive amination between the hemiacetal end group of HPMC and the amino end group of NH_2_-PLA. The resulting HPMC-PLA copolymers were able to self-assemble in an aqueous medium to form spherical micelles with a narrow distribution. Paclitaxel (PTX) was loaded in HPMC-PLA micelles. Higher drug loading was obtained with a higher PLA block length. A biphasic release profile was observed, i.e., an initial burst release followed by a slower release. PTX-loaded HPMC-PLA micelles exhibited significant toxicity to SK-BR-3 tumor cells, suggesting that these micelles could present great potential as nanocarriers of hydrophobic antitumor drugs.

**Figure 4:**
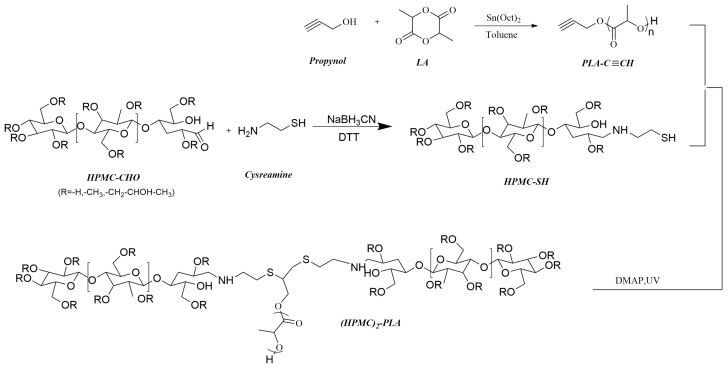
Synthesis route of PLA-(HPMC)_2_ block copolymers by reductive amination and click reaction [38].

Reductive amination could be used not only to couple polymers on the reducing end of polysaccharide, but also to functionalize the reducing end of polysaccharides. Polysaccharide-based copolymers can be constructed by coupling another block by click reaction or chain extension, with a functional polysaccharide acting as an initiator or a chain transfer agent. Click chemistry refers to reactions known for their exceptional efficiency, straightforward methodology, and versatility for reactions involving various functional groups. Coined by Sharpless in 2001, a “click” reaction is expected to have high yields, broad ranges of suitable substrates, facile product isolation, and tailorable characteristics [36]. On the other hand, click reactions involving polymers have an equimolar ratio of reactive groups and rapid reaction kinetics [37]. Lu et al. synthesized AB_2_-type PLA-(HPMC)_2_ block copolymers using a three-step procedure, as shown in Figure 4 [38]. In this process, alkynyl-terminated PLA was first obtained by the ring-opening polymerization of L-lactide initiated by propynol. In parallel, HPMC chain end was functionalized through reductive amination with 2-aminoethanethiol. These two end-functionalized polymers were subsequently coupled together using UV irradiation to trigger a thiol-ene click reaction. The resulting PLA-(HPMC)_2_ block copolymers exhibited self-assembly properties, forming micelles with diameters ranging from 50 to 100 nanometers. Importantly, the micelles displayed a relatively low critical micelle concentration (CMC) in the range of 0.14 to 0.16 g/L. Therefore, bio-based and biodegradable PLA-(HPMC)_2_ copolymers could be promising as nanocarriers of hydrophobic drugs.

As mentioned earlier, conventional synthetic block copolymers can be prepared using techniques such as atom transfer radical polymerization (ATRP), reversible addition-fragmentation chain transfer (RAFT), and nitroxide-mediated radical polymerization (NMRP) [39,40]. These methods allow for the achievement of good control over the molecular weight and dispersity of the resulting copolymers. In the case of polysaccharide-containing block copolymers, similar approaches have been employed with the addition of an initiator or a chain transfer agent to the reducing end of polysaccharides [41,42]. This allows for a chain extension, enabling the synthesis of block copolymers with significantly higher molecular weights compared to the coupling methods. In fact, coupling high-molecular-weight polymers together, especially those that diffuse relatively slowly, often proves to be slow and inefficient. To functionalize polysaccharides with an initiator, a combination of reductive amination and click chemistry is frequently used. This approach provides a versatile means of introducing the desired functionalities into the polysaccharide structure, paving the way for the subsequent synthesis of block copolymers [43].

Reductive amination is one of the simplest and widely used methods to construct polysaccharide-based block copolymers. Nevertheless, it also presents some limitations. First, the reaction kinetics can be rather slow. Reductive amination can be a time-consuming process due to the low availability of free aldehydes on the polysaccharide-reducing ends compared to the ring-closed hemiacetal form. This equilibrium can result in long reaction times up to several days. Second, the conversion rate can be incomplete. Equimolar reactions between polymer blocks, especially when they have moderate molecular weights, can lead to incomplete conversion due to the low concentration of the complementary reactive chain ends. Third, purification of the end products can be challenging. In fact, incomplete conversion in polymer coupling reaction requires purification steps to separate unreacted homopolymers from the desired diblock copolymer, which can be difficult as the solubility of the copolymer can be rather close to that of the starting blocks [29,43].

### 2.2. Oxime Ligation

Oxime ligation, known for its selectivity under mild conditions, has found widespread applications in the field of organic chemistry, particularly in bioconjugation and drug synthesis. This reaction proceeds efficiently at neutral or slightly acidic pH, which is compatible with a variety of biological systems without causing disruption to sensitive functional groups. Furthermore, oxime ligation has relatively fast reaction kinetics and versatility in accommodating various functional groups, especially those containing hydroxylamine moieties. The formation of stable oxime linkages ensures the stability of products and minimizes the risk of side reactions. Oxime ligation, with its advantageous features, plays a major role in the realms of chemical synthesis and biological research.

**Figure 5:**
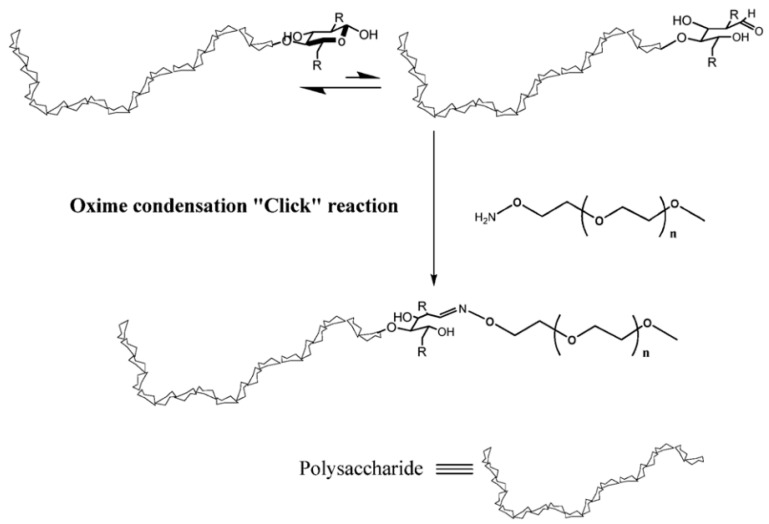
Synthesis of polysaccharide-PEG block copolymer by oxime ligation [44].

In 2012, Novoa-Carballal and Müller introduced a direct approach for the synthesis of block copolymers containing polysaccharides, utilizing oxime ligation by the reaction of an aldehyde and an aminooxy group, sometimes referred to as oxime click [44]. As shown in Figure 5, α-methoxy-ω-amino poly(ethylene glycol) (MeO–PEG–ONH_2_) was attached to the reducing end of a polysaccharide, yielding a polysaccharide–PEG block copolymer. One notable advantage of this approach is its utilization of the aldehyde group at the reducing end of the polysaccharide without the need for prior modifications. Oxime ligation with amine-functionalized PEG was successfully demonstrated with various polysaccharides, including dextran, chitosan, and hyaluronic acid. This approach allowed for the one-pot synthesis of diblock copolymers, eliminating the requirement for a catalyst, harsh reaction conditions, or intermediate products. Notably, oxime ligation was successfully applied to couple polysaccharides with molecular weights (Mn) exceeding 40 kg/mol and PEG with an Mn of 5 kg/mol. Although purification steps were necessary to isolate the desired block copolymer products, this approach holds promise for the development of block copolymers that may contain charged polysaccharide blocks. Such polymers could find utility in the creation of interpolyelectrolyte complexes for applications as protein and gene carriers. In another work, terminal primary alkoxyamine was coupled to the reducing end of a polysaccharide via oxime ligation to construct block copolymers [45].

**Figure 6:**
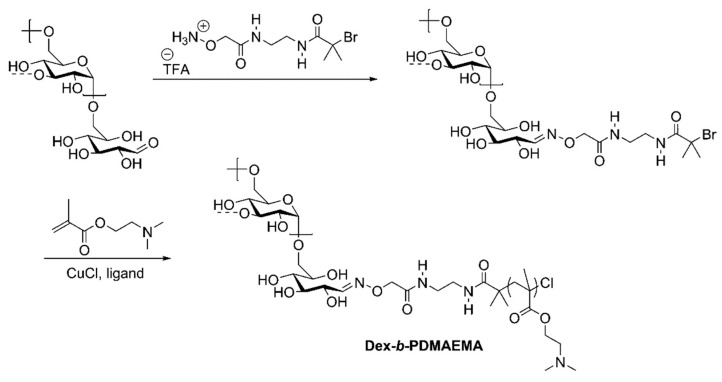
Synthesis of polysaccharide-containing block copolymers by oxime ligation of an ATRP initiator for chain extension [46].

Oxime ligation has been used for the synthesis of dextran-based copolymers by attaching an atom transfer radical polymerization (ATRP) initiator to initiate the growth of poly(2-(dimethylamino)ethyl methacrylate) (PDMAEMA) from the reducing end of dextran, as depicted in Figure 6. The ATRP reaction was conducted using various ligands and either CuBr or CuCl as a copper source, resulting in conversion rates ranging from 25% to 65% [46].

When compared to reductive amination, oxime ligation proves to be a more effective method in direct coupling reactions, facilitating the construction of larger block copolymers. This enhanced efficiency can likely be attributed to the higher nucleophilicity of hydroxylamines in comparison with amines, a phenomenon referred to as the alpha effect. However, it is noteworthy that the synthesis of oxime compounds is usually necessary because such derivatives are typically not commercially available. In addition, a limitation arises from the low concentration of polysaccharide aldehydes present at the reducing ends due to the equilibrium between hemiacetal and aldehyde forms. This limitation leads to long reaction times for oxime ligation reaction [43].

### 2.3. Other Chain Degradation—Nucleophilic Displacement 

**Figure 7:**
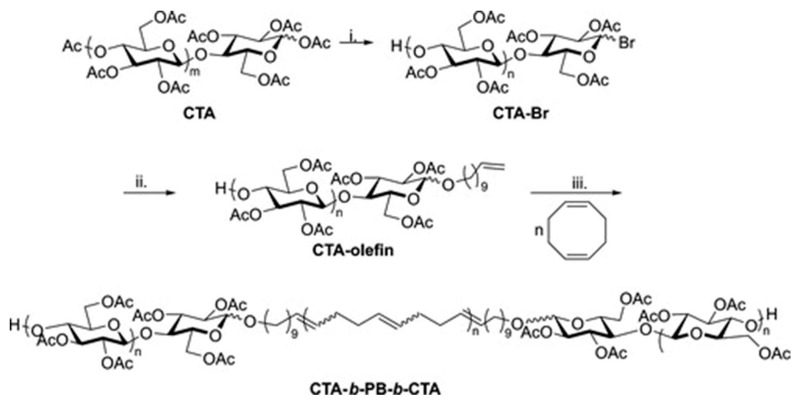
Synthesis of CTA-*b*-PB-*b*-CTA ABA triblock copolymer [47].

The Matson laboratory has introduced a method for the synthesis of ABA-type triblock copolymers containing polysaccharide segments [47]. This method involves modifying the end group of polysaccharides and utilizing ring-opening metathesis polymerization (ROMP). Cellulose triacetate (CTA) was used as the starting material. In this process, the anomeric linkages in commercial CTA first react with HBr, resulting in cellulose acetates with 1-bromo groups at the reducing end, as depicted in Figure 7. Subsequently, the bromide groups were replaced using a terminally unsaturated alcohol, yielding a CTA with mono-olefin functionalization. These terminal olefins (RHC = CH_2_, where R represents the substituted polysaccharide) were then employed as chain transfer agents in the ROMP of unhindered, strained cyclic olefins, thus incorporating functional groups (represented by R) at both ends of the polymer chains. This method enables the synthesis of ABA-type triblock copolymers with polysaccharide components by modifying the end groups of polysaccharides using ROMP. The approach is adaptable for various polysaccharide derivatives with suitable organic solubility.

## 3. Polysaccharides in the Construction of Nanocarriers

Polysaccharides and their derivatives are generally biodegradable and exhibit good biocompatibility with various tissues [48]. Polysaccharide-based copolymers, including grafted or brush-like copolymers and block copolymers, are widely used in the biomedical sector, especially as drug delivery systems.

The self-assembly of amphiphilic block copolymers into micelles has been extensively investigated. Micelles consist of a core–shell nanostructure obtained by the self-assembly of amphiphilic macromolecules in an aqueous medium. The hydrophobic component forms the core which is able to encapsulate hydrophobic drugs, whereas the hydrophilic component constitutes the shell providing the desired properties such as stabilization of the system and prolonged circulation by avoiding clearance. Micelles are typically 10–200 nm in diameter, enabling them to extravasate through the leaky vasculature in tumor tissue [49]. Compared with other long-circulating nanocarriers like nanoparticles or vesicles, polymeric micelles present many advantages, including controlled drug release by introducing stimuli-responsive structures on the polymeric chains, increased tissue penetrating ability, and reduced toxicity by introducing a functional group on the surface [50].

The use of polysaccharides as a building block in the construction of micelles presents many advantages as compared to synthetic polymers such as PEG and poly(N-isopropylacrylamide) (PNIPAAm), including low immunogenicity, compatibility with various tissues, and biodegradability [51,52,53]. Moreover, tailoring the functionalization of the polysaccharide backbone enables control over polymer solubility and responsiveness to stimuli such as pH or temperature. Many polysaccharides contain free hydroxyl groups, facilitating functionalization with carboxylic acid or hydroxyl-containing drugs like cholesterol [51,52,53]. Table 1 summarizes the various nanocarriers formed by polysaccharide-based amphiphilic block copolymers reported in the literature.

### 3.1. Cellulose

Cellulose is the major component of many plants. It is a linear β-1,4-glucan with a covalent acetal linkage between the C4 and C1 carbon atoms [65]. A cellulose macromolecular chain has one non-reducing end terminated with the original C4-OH group and a reducing end terminated with an original C1-OH group. Cellulose is insoluble in water and common organic solvents due to strong intermolecular and intramolecular hydrogen bonding. Degradation of macromolecular chains or modification of the backbone allows for the destruction of the network of hydrogen bonds, consequently yielding cellulose derivatives with improved solubility.

Cellulose can be quantitatively degraded by acid treatment [66] or by cellulase-catalyzed hydrolysis [67]. A great deal of hydroxyl groups can be esterified or etherified on the cellulose backbone, yielding various water-soluble cellulose derivatives such as cellulose acetate (CA), hydroxypropyl cellulose (HPC), hydroxypropyl methyl cellulose (HPMC), methyl cellulose (MC), hydroxyethyl cellulose (HEC), ethyl cellulose (EC), carboxy methyl cellulose (CMC), and hydroxyethyl methyl cellulose (HEMC). These derivatives, in particular EC, HPC [68], HPMC [68], and HEC [69], have been explored as a hydrophilic block to construct amphiphilic copolymers, which are able to self-assemble into micelles. In addition to polymer materials, nanocellulose-based hybrid organic/inorganic materials have also been reported for use in drug delivery systems [70,71].

**Figure 8:**
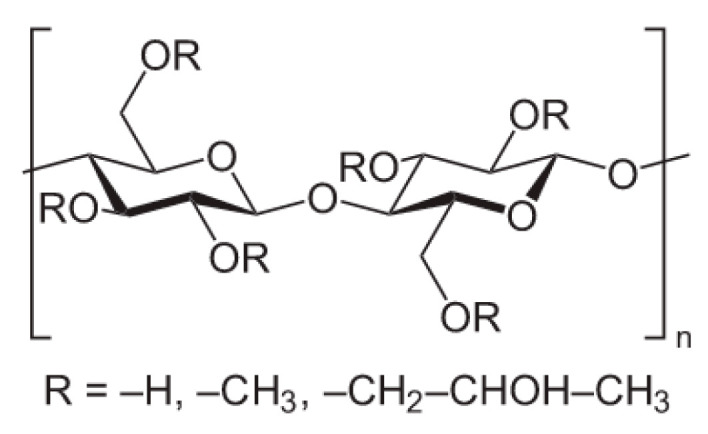
Chemical structure of HPMC. The substituent R represents a –CH_3_ group, a –CH_2_CH(CH_3_)OH group, or a hydrogen atom.

HPMC is a cellulose derivative resulting from the substitution of hydroxyl groups by hydroxypropyl and methyl groups. The chemical structure of HPMC is illustrated in Figure 8. The physicochemical properties of HPMC are strongly affected by the degree of substitution, molar degree of substitution, and degree of polymerization [72,73]. The degree of substitution refers to the average number of substituted hydroxyl groups, and the molar degree of substitution refers to the number of substituents introduced into the anhydroglucose unit [73,74].

HPMC is one of the most important hydrophilic biopolymers used for the preparation of oral controlled drug delivery devices due to its high swellability, which is the key characteristic determining drug release kinetics. Upon contact with water or biological fluid, the latter diffuses into the HPMC-based devices, leading to polymer chain relaxation with volume expansion [72]. Subsequently, the incorporated drug will be released by diffusion out of the system. HPMC-based hydrogels have been used as carrier for several drugs such as naproxen, tramadol, and ibuprofen [75]. The factors influencing drug release behaviors have been investigated, as documented in a review by Kamel et al. [75]. A composite of HPMC with indomethacin, an anti-inflammatory drug, was formulated by the supercritical fluid-assisted impregnation method [76]. Data show that drug release obeys a power law (n = 0.54). This strategy is very promising because it allows for the preparation of natural drug carriers in a ‘green’ way. HPMC is also used to form thermo-sensitive reversible hydrogels based on hydrophobic interactions [73].

Wang et al. prepared amphiphilic HPMC-PLLA diblock copolymers by a UV-initiated thiol-ene click reaction [54]. Thiol-terminated HPMC (HPMC-SH) was obtained by coupling the reducing end group of HPMC with the amine group of cysteamine, followed by reductive scission of the central disulfide bond of the resulting HPMC-S-S-HPMC by using excessive DL-1,4-dithiothreitol (DTT). Meanwhile, allyl-terminated PLLA was prepared by the ROP of L-lactide in the presence of allyl alcohol. Subsequently, a UV-initiated thiol-ene click reaction was performed to couple HPMC-SH with allyl-terminated PLLA, yielding a HPMC-PLLA diblock copolymer. The resulted amphiphilic copolymers are water-soluble and able to self-assemble in micelles in an aqueous medium. The micelle size increases with the increasing molecular weight of the HPMC hydrophilic block. The HPMC block length also affects the critical micelle concentration (CMC) and the lower critical solution temperature (LCST) of the copolymers. Both the CMC and LCST increase with increasing HPMC block length. These results highlight the importance of the hydrophilic block or hydrophilic/hydrophobic balance on the self-assembly behavior of HPMC-PLA block copolymers, which could be very promising as nanocarriers of hydrophobic drugs.

Other cellulose derivatives have also been considered for the construction of drug carriers, especially cellulose acetate (CA) and carboxymethyl cellulose (CMC). CA is a class of derivatives with the substitution of hydroxyl groups by acetate ones. Studies mainly focus on CA beads and electrospun fibers for the encapsulation of dyes and drugs. Electrospinning has attracted great interest for biomedical usages in recent years. Electrospun nanofibers provide many advantages for drug delivery applications such as high flexibility, enhanced control over drug release kinetics, simultaneous delivery of different drugs, and enhanced local therapeutic effects [77]. Moreover, various drug-loading methods can be employed by using different electrospinning methods with a high encapsulation efficiency and loading capacity [78]. For example, multiaxial electrospinning is used for entrapment of the therapeutic agents into core–shell nanofibers or multilayered fibers. These structures allow a premature release to prevent barrier and prolonged drug release [79,80]. Electrospun CA nanofibers are widely applied for the encapsulation of therapeutic agents such as antimicrobial, antibacterial, antioxidant, and anti-inflammatory agents. Recently, research has focused on uses of electrospun CA nanofibers in topical/transdermal drug delivery systems. CA-based stimuli-responsive drug delivery systems have also been developed [81].

Carboxymethyl cellulose (CMC) is a class of derivatives with negative charges on the polymer chains. Therefore, CMC is able to self-assemble together with positively charged polymers or proteins to yield coacervates as drug nanocarriers [81]. In fact, CMC has been used in a number of drug delivery and tissue-engineering purposes. Apomorphine, a drug clinically used to regulate motor response in Parkinson’s disease, was successfully loaded in a CMC powder formulation. A sustained nasal release was observed [82]. CMC-encapsulated layered double hydroxides/drug nanohybrids have been studied for the oral delivery of cephalexin [15]. Sodium CMC has been applied in gastrointestinal drug delivery [83]. CMC is considered as a promising drug carrier for release in buccal tissue [84].

**Figure 9:**
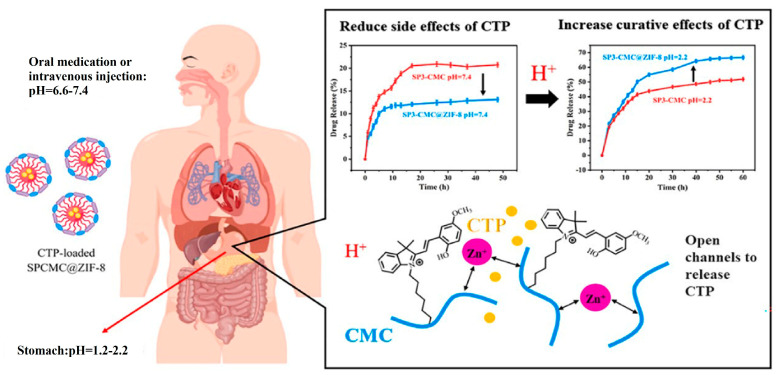
Combination of NaCMC-based micelles and ZIF-8 metal–organic frameworks as acid-responsive drug carrier [86].

A number of CMC-based copolymers have been reported. CMC-*g*-PLA graft copolymers were synthesized by the ROP of lactide and used as nanocarriers of doxorubicin (DOX) [85]. Additionally, an anti-EpCAM antibody, specific to EpCAM protein overexpressed on hepatocytes, is attached to CMC for targeted drug delivery. In aqueous environment, CMC-*g*-PLA was able to self-assemble into micelles and encapsulate DOX in the hydrophobic core of PLA. DOX-loaded micelles exhibited pH-induced drug release due to the faster degradation of PLA at an acid pH as compared to a neutral environment. The micelles entered cancer cells through endocytosis under acidic conditions, enhancing the therapeutic efficacy. Recently, sodium CMC (NaCMC)-based micelles and ZIF-8 metal–organic frameworks (MOFs) are combined to construct acid-responsive nanocarriers of camptothecin (CPT), a hydrophobic antitumor agent [86]. CPT is coated on the core of micelles by hydrophobic interaction with spiropyran (SP), a pH-responsive compound used as an additive in drug release devices, and ZIF-8 grows on the surface of the micelle to reduce drug leakage. In acidic conditions, ZIF-8 collapse provides zinc ions as crosslinking agents for NaCMC, improving the drug release as compared to SP-grafted NaCMC micelles. The intrinsic biological adhesion of NaCMC ensures sustained drug release for up to 40 h (Figure 9). Folate-decorated CMC was also used to prepare amphiphilic graft copolymers for loading DOX [87].

### 3.2. Hyaluronic Acid

Hyaluronic acid (HA) is a naturally charged glycosaminoglycan composed of D-glucuronic acid and N-acetyl-d-glucosamine alternating on the backbone connected by β-1,4 and β-1,3 glycosidic linkages. HA is biocompatible, non-toxic, non-immunogenic, non-inflammatory, and degradable by native enzymes [88]. Thus, it has been used for various medical applications including arthritis treatment, wound dressing, ocular surgery, and tissue regeneration. The molecular weight of HA is crucial in the formulation of pharmaceutical products to achieve specific biological effects. Different from native HA, HA fragments exhibit diverse effects on inflammation, angiogenesis, fibrosis, cancer, and autoimmune response [89,90]. HA also has received much attention as a biopolymer for the development of drug delivery systems, including ocular and nasal delivery systems, and sustained release formulations via subcutaneous injection. Furthermore, the affinity of HA to the CD44 receptor, which is overexpressed in various tumor cells, makes HA an important biopolymer in cancer-targeted drug delivery.

**Figure 10:**
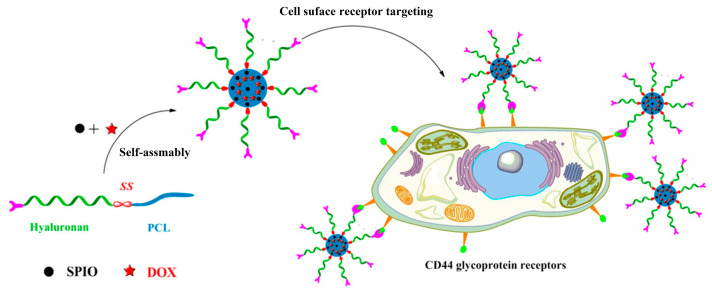
HA-ss-PCL nanocarriers for chemotherapy and MR imaging [55].

Nanocarriers prepared from HA-based amphiphilic block copolymers, including prodrug, dendrimers, or micelles, can not only reduce phagocytosis by the reticuloendothelial system (RES), but also achieve active targeting of tumor cells due to the overexpression of CD44 [91,92,93]. Jiang et al. prepared theranostic nanocarriers by the self-assembly of HA-based block copolymers for chemotherapy and MR imagining, as shown in Figure 10 [55]. Redox-sensitive and active targeting HA-ss-PCL block copolymers were synthesized by successive ROP, reductive amination, and click reaction. These copolymers could form spherical micelles with average sizes between 83 and 193 nm. The micelles showed a high loading capacity for both DOX and superparamagnetic iron oxide (SPIO) and exhibited a quicker release of DOX in the presence of 10 mM glutathione (GSH). MTT assay and flow cytometry results demonstrated that DOX-loaded HA-SS-PCL micelles enhanced cellular uptake and cytotoxicity against HepG2 cells compared to normal HA-PCL micelles. Additionally, SPIO-loaded HA-SS-PCL micelles exhibited higher MRI sensitivity and relaxivity, showing significant potential as actively targeted, redox-sensitive theranostic nanocarriers for the diagnosis and chemotherapy of hepatic carcinoma.

HA-based polymeric micelles were also studied as carriers for photodynamic cancer therapy [56]. HA-PLGA block copolymer was synthesized by the end-to-end coupling of HA-NH_2_ and PLGA-COOH. Protoporphyrin IX (PpIX), a potent photosensitizer, was loaded in HA-PLGA micelles via dialysis. The micelles exhibited a particle size of approximately 200 nm, and the drug encapsulation efficiency exceeded 43%. Significantly enhanced phototoxicity toward CD44-overexpressing A549 cells was observed in both the 2D monolayer cell culture and 3D tumor spheroids. This enhanced effect was attributed to the promoted cell uptake and deeper penetration of the micelles into the spheroids. Jiang et al. reported the synthesis of an amphiphilic galactosamine–hyaluronic acid–vitamin E succinate (Gal-HA-VES) block copolymer [57]. Multifunctional micelles were prepared for the delivery of norcantharidin (NCTD) to a hepatic carcinoma. NCTD-loaded Gal-HA-VES showed higher cytotoxicity toward CD44- and ASGP-R-overexpressing cells, in agreement with the enhanced cellular uptake. A cell apoptosis assay indicated that NCTD-loaded Gal-HA-VES micelles were more effective in triggering apoptosis, compared with free NCTD or NCTD-loaded HA-VES micelles. In vivo tests demonstrated that NCTD-loaded Gal-HA-VES micelles exhibited enhanced tumor targeting and antitumor activity with lower systemic toxicity, suggesting that they can achieve significant tumor targeting and effective treatment of hepatic carcinoma.

### 3.3. Dextran

Dextran is a representative of neutral polysaccharides and primarily comprises α-1,6-linked glucopyranoside units with small quantities of α-1,2-, α-1,3-, and α-1,4-branched chains [94]. It is generally synthesized by lactic acid bacteria, but a commercially available biopolymer is typically derived from sucrose-containing sources. The hydroxyl groups present on the dextran backbone allow for easy functionalization. Moreover, dextran exhibits biodegradability, biocompatibility, hydrophilicity, non-toxicity, and stability within the bloodstream. Dextran has also demonstrated anti-thrombotic and anti-inflammatory properties.

Dextran has found various applications, including the preparation of hydrogels [95,96], micelles [96,97], and nanoparticles [98]. The use of grafted dextran for micelle formation has been extensively explored, particularly in the context of anticancer drug delivery [99,100]. Lipids such as cholesterol, oleic acid, and stearic acid have been conjugated with dextran, yielding amphiphilic polymers conducive to efficient micellization and entrapment of chemotherapeutic agents [101,102,103]. For instance, PTX-loaded dextran-stearate micelles were developed for breast cancer therapy, demonstrating the potential of dextran–stearate micelles for intracellular DOX delivery [101].

A number of dextran-based amphiphilic block copolymers have also been synthesized by attaching a hydrophobic block such as polystyrene [32,104], PCL [105,106,107], PLA [108,109,110], poly(alkyl cyanoacrylate) [111], polypeptides [112], and deoxycholic acid polyesters [58]. Nichifor et al. synthesized copolymers by dipolar 1,3-cycloaddition reaction between dextran with azide end groups and deoxycholic acid—oligo(ethylene glycol)s polyester with propargyl end groups [58]. Different copolymer compositions were obtained by varying the molecular weights of dextran (Mn 4.5, 8, 15 kDa) and polyester (Mn 2–6 kDa). Micelle-like aggregates were obtained by the self-assembly of copolymers in an aqueous medium with nanometric sizes (50–600 nm) and spherical forms. Curcumin, a hydrophobic antitumor drug, was encapsulated in micelles with greatly enhanced water solubility. Slow curcumin release from micelles was observed with a reduced burst effect. A star-shaped copolymer, sPCL-dextran, was also studied as a drug delivery system [59]. The copolymer consists of a dipentaerythritol core, with a hydrophobic PCL inner arm block and a hydrophilic dextran outer arm block.

**Figure 11:**
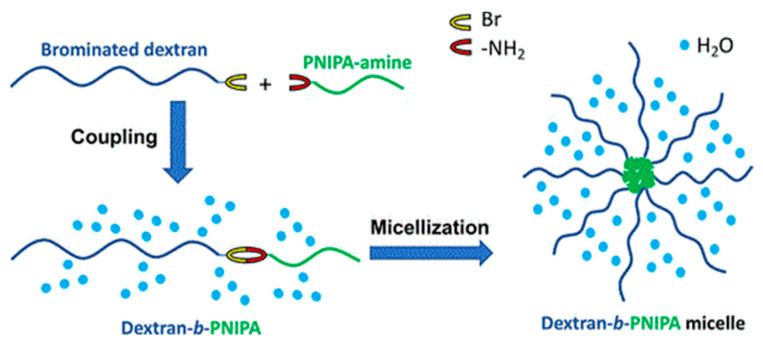
Schematic view of the synthesis and micellization of DEX-*b*-PNIPA block copolymer [60].

Edgar et al. reported a new route to synthesize dextran-based block copolymers by regioselective bromination of the dextran non-reducing end, as shown in Figure 11 [60]. The regioselective bromination of the exclusive primary alcohol group at the C-6 position of the non-reducing end was achieved through the use of N-bromosuccinimide (NBS) and triphenyl phosphine (PPh3). The functionalized dextran was subsequently utilized in the synthesis of block copolymers with various amine-terminated polymers, leading to the formation of diverse block copolymers such as DEX-*b*-PEG, DEX-*b*-polystyrene (DEX-*b*-PS), and DEX-*b*-poly(N-isopropylacrylamide) (DEX-*b*-PNIPA). The latter exhibits thermally induced micellization, with micelle formation occurring above 33 °C. 

### 3.4. Chitosan

Chitosan is a N-deacetylated product of chitin, the second-most abundant polysaccharide in nature [113]. On the chitosan chain, there are amounts of amino and hydroxyl groups, which can act as reaction points for combinations with crosslinkers, target ligands, or drugs. Chitosan is soluble in mildly acidic media due to the presence of amino groups and is pH-responsive as the amino groups can be converted to ammonium ones [114]. As a natural cationic polymer, chitosan has been explored to construct drug delivery systems due to its low immunogenic, biocompatible, and hydrophilic properties [115,116]. In addition, chitosan is readily biodegradable in vivo by several enzymes [117]. Nevertheless, like other biopolymers, chitosan has poor water solubility due to strong hydrogen bonding. The water solubility of chitosan can be improved by introducing hydrophilic groups [48,118] or by degradation into short chains [119]. In general, chitosan with a lower molecular weights and a higher degree of deacetylation demonstrates increased solubility and faster degradation compared to chitosan with a higher molecular weight and lower degree of deacetylation [120].

Chitosan particles prepared via the coacervation method were used to encapsulate genetic material for applications in gene therapy. Coacervation between positively charged amine groups on chitosan and negatively charged phosphate groups on DNA facilitates the formation of chitosan–DNA nanoparticles, as reported in the literature [121]. The system could efficiently protect the genetic material from a nuclease attack. The weight ratio of the two polymers significantly influences the particle size, surface charge, entrapment efficiency, and release characteristics of the nanoparticles. The transfection efficiency is dependent on the molecular weight of chitosan, concentration of nucleotide, and type of cells [122,123]. Hydrogels based on chitosan have been used as drug carriers in the field of cancer treatment, using various methods of preparation and crosslinking agents. Entrapped drugs included PTX, DOX, and CPT [124].

Several studies have focused on the development of chitosan-based micelles through modification of the backbone structure. Efficient drug delivery vehicles have been obtained from lipid-modified chitosan [125,126]. Another approach involved the conjugation of PEGylated chitosan with lipoic acid, forming a drug delivery platform [127]. Chitosan was also modified with cholesterol to prepare a drug delivery system for poorly soluble anticancer drugs [126]. Palmitic acid was utilized to functionalize the amine groups in chitosan for the delivery of a hydrophobic drug, tamoxifen [128]. The resulted nanocarrier demonstrated efficient drug encapsulation, sustained drug release, and hemocompatibility. Polymer-grafted chitosan has also been investigated for drug delivery applications [129,130]. For instance, chitosan-*g*-PLA was synthesized through the ROP, and nanomicelles were prepared from the graft copolymer to load a hydrophobic antioxidant, β-carotene [129].

Chitosan-based block copolymers have also been reported. An amphiphilic chitosan-*b*-poly(p-dioxanone) (CS-*b*-PDO) block copolymer was synthesized by intermolecular nucleophilic reaction between free-radical-containing chitosan and a carbon–carbon double bond-containing PDO-macromer [61]. The copolymer was capable of self-assembling in neutral aqueous solutions and partially disassembling in acidic endosomal/lysosomal environments. Copolymer micelles were utilized to encapsulate CPT, an antitumor drug. In vitro drug release studies revealed a significantly faster CPT release at pH 5.0 compared to pH 7.4. Blank micelles demonstrated non-toxicity in preliminary cytotoxicity assays. Cell experiments confirmed the effective internalization of CPT-loaded micelles by Hela cells, leading to potent antitumor efficacy. Another chitosan-based block copolymer, N-succinyl-N’-4-(2-nitrobenzyloxy)-succinyl-chitosan was synthesized to develop near-infrared (NIR) light-breakable micelles [131]. By encapsulating an NIR dye cypate into the hydrophobic core of micelles, the dissociation of micelles under NIR exposure could trigger the release of co-loaded hydrophobic species [62]. The micelles formed from a chitosan-co-poly(ethylene glycol) methyl ether methacrylate block copolymer present a promising strategy for hydrophobic drug delivery [63]. Their pH-responsive swelling mechanism, driven by chitosan protonation, facilitates a controlled drug release. The polymeric micelles, with a protective PEG corona, resist protein adsorption and extend circulation times. Sized between 50 and 350 nm, these stable micelles offer a potential solution for enhanced and prolonged drug administration.

### 3.5. Alginate

Alginate is a type of anionic hydrophilic polysaccharide primarily derived from brown algae and some soil bacteria [132]. It consists of linear chains of alternating residues of two monosaccharides: α-L-guluronic acid (G-blocks) and β-d-mannuronic acid (M-blocks). The composition and sequence of G and M blocks, including GG, MM, MG, and GM blocks, can vary to a large extent, leading to different molecular weights and physical properties of alginates. Alginates can have molecular weights ranging from 10 to 1000 kDa [132]. The versatility of alginate-based materials in terms of structure, functionality, and ability to carry different active substances makes them very attractive for a wide range of medical and pharmaceutical applications [133,134].

Alginate-based drug delivery systems have been developed, including hydrogels, nanoparticles, microparticles, liposomes, and tablets. Thermo-sensitive hybrid polymer complex micelles were prepared from sodium alginate-*g*-poly(N-isopropyl acrylamide) (SA-*g*-PNIPAM) and divalent metal ions to encapsulate DOX [135] and 5-fluorouracil (5-FU) [136]. The micelles were spherical in shape and exhibited excellent drug encapsulation performance. The cumulative drug release from micelles was controlled by pH, ionic strength, or temperature of the medium. An ABC-type triblock copolymer alginate-*b*-PEG-*b*-PLA was synthesized by the covalent attachment of alginate with a PLA-PEG diblock copolymer [64]. These amphiphilic block copolymers self-assembled into nanoparticles in an aqueous medium and could encapsulate both hydrophobic and hydrophilic payloads. A spatiotemporal release of the co-formulated drug payloads was observed, showing promise as a co-delivery system for combination therapies.

## 4. Conclusions and Perspective

The utilization of polysaccharides as hydrophilic building blocks in the construction of amphiphilic block copolymers presents great interest in the realm of drug delivery systems. The advantages of employing polysaccharide-based copolymers, including their biocompatibility, versatility, and tunable properties, underscore their significance in enhancing the performance of nanocarriers. Thanks to the presence of a reducing end in polysaccharide chains, various amphiphilic block copolymers can be constructed from polysaccharides and hydrophobic polymers bearing amine end groups. The exploration of various nanocarrier structures, such as nanogels, nanoparticles, and micelles, further expands the potential applications of polysaccharide-based systems in delivering therapeutic agents with precision and efficiency. The synthesis methods discussed, encompassing reductive amination, oxime ligation, and other chain-end modification reactions, offer a toolkit to tailor the structure properties of copolymers for specific applications. The description of specific polysaccharides, including cellulose, hyaluronic acid, dextran, alginate, and chitosan, highlights their unique attributes as hydrophilic blocks to construct amphiphilic block copolymers. The showcased examples demonstrate the feasibility of the construction of polysaccharide-based nanocarriers for controlled or targeted drug delivery. Future research could explore novel polysaccharides or combination strategies that enhance the performance and functionality of nanocarriers. Additionally, advancements in understanding the interactions between nanocarriers and biological systems will be crucial for optimizing drug delivery efficiency and minimizing potential side effects. Therefore, the development of polysaccharide-based nanocarriers holds great potential for further innovations and breakthroughs, opening new avenues in drug delivery and nanomedicine. 

## Figures and Tables

**Table 1:** Nanocarriers formed by polysaccharide-based amphiphilic block copolymers.

Polysaccharide	Block Copolymer	Nanocarrier	Loaded Drug	References
HPMC (cellulose derivative)	HPMC-PLLA	micelles	-	[54]
HPMC (cellulose derivative)	HPMC-PLA	micelles	Paclitaxel	[33]
HPMC (cellulose derivative)	HPMC-JEF	micelles	-	[34]
HPMC (cellulose derivative)	HPMC-PCL	micelles	Curcumin	[35]
Hyaluronic acid	HA-ss-PCL	micelles	DOX, superparamagnetic iron oxide (SPIO)	[55]
Hyaluronic acid	HA-PLGA	micelles	Protoporphyrin IX (PpIX)	[56]
Hyaluronic acid	Gal-HA-VES	micelles	Norcantharidin (NCTD)	[57]
Dextran	Dextran-deoxycholic acid polyesters	micelles	Curcumin	[58]
Dextran	sPCL-dextran	micelles	-	[59]
Dextran	DEX-*b*-polystyrene (DEX-*b*-PS), DEX-*b*-poly(N-isopropylacrylamide) (DEX-*b*-PNIPA)	micelles	-	[60]
Chitosan	CS-*b*-PDO	micelles	CPT	[61]
Chitosan	N-succinyl-N’-4-(2-nitrobenzyloxy)-succinyl-chitosan	micelles	Fluorescence dyes, (fluorescein and cypate)	[62]
Chitosan	chitosan-co-poly(ethylene glycol)	micelles	Fluorescein	[63]
Alginate	alginate-*b*-PEG-*b*-PLA	Nanoparticle	Rhodamine B, Azathioprine, Doxorubicin, Erlotinib, Irinotecan, Coumarin6	[64]

## Data Availability

Not applicable.

## Abbreviations

HAMA	N-(2-hydroxypropyl)methacrylamide
PLA	polylactide
PCL	poly(ε-caprolactones)
PGA	polyglycolide
PDO	polydioxanone
PLGA	poly(lactide-co-glycolide)
Dex	dextran
PS	polystyrene
HPMC	hydroxypropyl methyl cellulose
ROP	ring-opening polymerization
Boc	N-tert-butoxycarbonyl
PTX	paclitaxel
CMC	critical micelle concentration
ATRP	atom transfer radical polymerization
RAFT	reversible addition-fragmentation chain transfer
NMRP	nitroxide-mediated radical polymerization
Mn	molecular weights
PDMAEMA	poly(2-(dimethylamino)ethyl methacrylate)
ROMP	ring-opening metathesis polymerization
CTA	cellulose triacetate
CA	cellulose acetate
MC	methyl cellulose
HEC	hydroxyethyl cellulose
EC	ethyl cellulose
CMC	carboxy methyl cellulose
HEMC	hydroxymethyl cellulose
DTT	DL-1,4-dithiothreitol
LCST	lower critical solution temperature
DOX	doxorubicin
MOF	metal–organic frameworks
CPT	camptothecin
SP	spiropyran
HA	hyaluronic acid
RES	reticuloendothelial system
SPIO	superparamagnetic iron oxide
GSH	glutathione
PpIX	protoporphyrin IX
Gal-HA-VES	galactosamine-hyaluronic acid-vitamin E succinate
NCTD	norcantharidin
NBS	N-bromosuccinimide
PPh3	triphenyl phosphine
DEX	dextran
PNIPA	poly(N-isopropylacrylamide)
CS-b-PDO	chitosan-b-poly(p-dioxanone)
NIR	near-infrared
SA-g-PNIPAM	sodium alginate-g-poly(N-isopropyl acrylamide)
5-FU	5-fluorour
